# Acquired long QT syndrome in chronic kidney disease patients

**DOI:** 10.1080/0886022X.2019.1707098

**Published:** 2019-12-27

**Authors:** Peng Liu, Lu Wang, Dan Han, Chaofeng Sun, Xiaolin Xue, Guoliang Li

**Affiliations:** a Department of Cardiovascular Medicine, First Affiliated Hospital of Xi’an Jiaotong University, Xi’an, P.R. China;; b Department of Endocrinology, First Affiliated Hospital of Xi'an Jiaotong University, Xi'an, P.R. China

**Keywords:** Chronic kidney disease, acquired LQT syndrome, QT interval, sudden cardiac death

## Abstract

Cardiovascular disease (CVD) is the leading cause of morbidity and mortality in chronic kidney disease (CKD) patients. QT interval prolongation is a congenital or acquired condition that is associated with an increased risk of torsade de pointes (TdP), sudden cardiac death (SCD), and all-cause mortality in the general population. The prevalence of acquired long QT syndrome (aLQTS) is high, and various acquired conditions contribute to the prolonged QT interval in patients with CKD. More notably, the prolonged QT interval in CKD is an independent risk factor for SCD and all-cause mortality. In this review, we focus on the epidemiological characteristics, risk factors, underlying mechanisms and treatments of aLQTS in CKD, promoting the management of aLQTS in CKD patients.

## Introduction

The QT interval represents electrical depolarization and repolarization of the ventricles. QT interval prolongation is a congenital or acquired condition that is associated with an increased risk of TdP and sudden cardiac death (SCD) in the general population. The prevalence of acquired long QT syndrome (aLQTS) is high and increases with the decline in kidney function in CKD patients [1–8]. The QTc interval increases by an average of 2.9 ms for each milligram increase in serum creatinine [3]. The risk of QTc prolongation is 1.20 times, 2.47 times and 3.35 times higher in CKD4, CKD5 and hemodialysis patients, respectively, than in CKD3 patients. CKD can cause a series of poor conditions, such as water-electrolyte disturbance, metabolism disorder, and uremic toxin accumulation, which can cause direct or indirect damage to the cardiovascular system. CKD is also known to impair drug disposition of renal eliminated QT-prolonged medications that may lead to unintended toxicity despite dose adjustment according to the glomerular filtration rate (GFR) [9]. Thus, various factors contribute to aLQTS in CKD. The prevalence of all-cause and cardiovascular mortality is significantly increased in CKD patients with aLQTS [2,4,5,10–14]. 

### QTc interval and aLQTS

The QT interval normally varies with heart rate. In this regard, many attempts (Bazett, Fridericia, Framingham and Hodges) have been made to ‘correct’ the QT interval to a value that might be expected if the heart rate is 60 beats per minute. The commonly used correction formulas are the Bazett, Fridericia, Framingham, Hodges, and Rautaharju formulas. The comparison of the superiority among different kinds of correction formulas showed that the Fridericia and Framingham formulas had the best correction, but the Bazett formula was the worst [15]. How long is too long for QTc? Interpretation of the ECG states that a QTc ≥450 ms (males) or ≥460 ms (females) is considered a prolonged QT interval. A QTc interval ≥500 ms is associated with a significantly increased risk of life-threatening cardiac events in adulthood [16]. QT interval prolongation can lead to TdP, which can result in SCD. For each 10 ms increase in the QT interval, there is a 5–7% increase in the risk of developing TdP, and every 20 ms increase in QT substantially increases the risk of TdP [17]. The acquired pathologic conditions that lead to QT prolongation and TdP are complex and diverse. Whereas prescription drugs account for the majority of cases of aLQTS, other causes include postmyocardial infarction QT prolongation, electrolyte disturbance, and intracranial bleeding (Table 1) [18–22,36,38–43].

**Table 1.** Risk factors for long QT syndrome.

Risk factors for long QT syndrome	References	Results
Age	[18,19]	Longer QTc was associated with increasing age.
Female gender	[19]	Female is a risk factor for QTc prolongation.
Smoking	[18]	Smoking is a risk factor for QTc prolongation.
Body mass index	[18]	High body mass index is associated with prolonged QT interval.
Electrolyte disturbances		
Hypokalemia	[18,19]	Hypokalemia is a risk factor for QT prolongation.
Hypocalcemia	[18]	Calcium treatment in patients with hypocalcemia can significantly short the repolarization interval and reduce the number of ventricular premature complexes.
Hypochloremia	[20]	Hypochloremia is related with QT prolongation.
Hyponatremia	[20]	Hyponatremia is related with QT prolongation.
Drugs	[21–26]	The risk of drug-induced QTc interval prolongation varies by drug and presence of risk factors
Comorbidities		
Cardiomyopathy;	[18,19]	Cardiomyopathy is a risk factor for QT prolongation.
Congestive heart failure;	[27,28]	Patients with severe systolic HF had statistically significant prolongation of the QTc interval. The prevalence of LQTS was 63% in patients with HF against 4.4% in normal populations
Left ventricular hypertrophy;	[29,30]	Myocardial hypertrophy induced by hypertension can result in ‘reduced repolarization reserve’, and thus a latent acquired LQTS
Diabetes;	[31,32]	The LQTS shows high prevalence in diabetic patients and it could forecast cardiovascular and all-cause death
Chronic kidney disease;	[22,33,34]	QT interval prolongation is prevalent in CKD and hemodialysis patients
Liver failure;	[20]	Liver cirrhosis is a risk factor for QT prolongation. QT prolongation is parallel with the severity of liver dysfunction.
Autonomic dysfunction;	[35]	Cardiovascular autonomic neuropathy was associated with prolongation of QT interval
Cerebrovascular accident	[36]	Electrocardiograph abnormalities are common in intracerebral hemorrhage The most frequent was ST depression, followed by left ventricular hypertrophy, QTc prolongation, and T wave inversion.
Depression	[29]	Depression is associated with QT prolongation.
Pulmonary disorders	[29,37]	Pulmonary disorders is associated with QT prolongation.
Thyroid disturbances	[34]	High free thyroxine levels are associated with QTc prolongation in male.

CKD: chronic kidney disease; LQTS: long QT syndrome.

**Table 2.** The prevalence and outcomes of long QT syndrome in CKD patients.

First author and year	Patients and follow-up	Results
Nappi et al. [4]	23 ESRD patients were treated with different Ca^++^ concentrations dialysate.	The QTc interval were measured before and after the three sessions. QTc increased after dialysis, which related with low Ca^++^ concentrations dialysate (1.25 mmol/L).
Beaubien et al. [14]	147 patients on maintenance HD (*N* = 49) or peritoneal (*N* = 98) dialysis. 5–9 years of follow-up.	A prolonged QTdc (>74 ms) was detected in 46.9% and 52% of HD and peritoneal dialysis patients, respectively. QTdc was associated with the presence of diabetes mellitus, mean QT interval, corrected calcium levels. QTdc was an independent predictor of total (RR = 1.53) and CV mortality (RR = 1.57).
Maule et al. [33]	69 ERSD patients and 12 subjects with normal renal function.	Compared to controls, ESRD patients showed a longer QTc (*p* = .016). After the HD session, QTc increased in 56% and decreased in 43% of the patients.
Familoni et al. [2]	42 patients on hemodialysis and 45 control subjects.	The prevalence of prolonged QTc was higher in dialysis patients compared with control subjects (*p* < .05). The maximum QTc was longer than 440 ms in 71.4% of patients post-dialysis. The in-hospital mortality was not statistically different between the prolonged QTc group (73.3%) and normal group (66.7%) in hemodialysis patients.
Kestenbaum et al. [13]	3238 participants with and without CKD. 9.2 years of follow-up.	Participants with CKD had longer QTc intervals compared with those without CKD. Each 5% increase in QTI was associated with a 42% (95% CI 1.23–1.65), 22% (95% CI 1.07–1.40) and 10% (95% CI 0.98–1.22) greater risk of HF, CHD and mortality, respectively.
Patane et al. [1]	Study present a case of torsade de pointes in an 82-year-old Italian woman with chronic renal failure.	It reported that (QTc interval prolongation and torsade de pointes are associated with ESRD and that they can be a cause of SCD in ESRD.
Hage et al. [10]	280 ESRD patients evaluated for transplantation. 40 ± 28 months of follow-up.	39% of patients exhibited a prolonged QTc (460 ms). Patients with a prolonged QTc (39%) had 1-, 3-, and 5-year death-rates of 12%, 36%, and 47%, respectively, vs 8%, 24%, and 36% for those with normal QTc (*p* = .03). The prolonged QTc was an independent predictor of mortality in ESRD patients (HR: 1.008)
Genovesi et al. [7]	122 patients undergoing HD were studied. 3.9 years of follow-up.	44 patients (36.0%) had a prolonged QTc. 51 patients died (41.8%), of whom 12 died for SCD. In multivariate analysis, prolonged QTc (HR = 2.16) were independently associated with total mortality and SCD.
Khosoosi et al. [5]	58 patients with chronic renal disease on chronic HD. The QTc was assessed 30 minutes before and after HD.	The mean of corrected QTc intervals increased significantly from 423.45 ± 24.10 to 454.41 ± 30.25 ms (*p* < .05). The changes in serum potassium and calcium levels were related with QT interval prolongation.
Flueckiger et al. [11]	930 adult ESRD patients evaluated for renal transplantation. 3.1 years of follow-up.	456 patients (49%) had a prolonged QTc. 108 (11.6%) patients died. Patients with 2 or more ECG interval prolongations had a 2.5-fold increased likelihood of dying vs. patients with no ECG interval prolongations (HR 2.53).
Sherif et al. [3]	154 CKD patients without structural heart disease or medications that are known to prolong QT interval.	QTc interval prolongation was present in 63.6% of the cases (63% of females and 64% of males), and severely prolonged (>500ms) in 19.5% (19.4% of females and 19.6% of males). QTc interval was significantly prolonged across increasing stages of CKD severity (*p* < .006).
Malik et al. [12]	Study followed 6565 participants with and without CKD. 13.3 years of follow-up.	CKD group had prolonged QTc than those without CKD (20.5%vs12.9%, *p* < .0001). Both prolonged QTc and CKD are independently associated with increased risk of mortality. When combined, risk of mortality is higher in those with CKD by eGFR with prolonged QTc than normal QTc (HR 2.6) and (HR 3.1) vs (HR 1.4) and (HR 1.7) for all-cause and CV mortality.
Liu et al. [8]	The prevalence of aLQTS was evaluated in 804 CKD patients.	The prevalence of aLQTS is much higher and increases with the decline of kidney function in hospitalized CKD patients, which is related to older age, impaired kidney function, hemodialysis, serum potassium and low LVEF.

LQTS: acquired long QT syndrome; CHD: coronary heart disease; CKD: chronic kidney disease; CV: cardiovascular; ECG: electrocardiograph; eGFR: estimated glomerular filtration rate; ESRD: end-stage renal disease; HD: hemodialysis; HF: heart failure; HR: hazard ratio; LVEF: left ventricular ejection fraction; QTc: corrected QT; QTd: QT dispersion; QTdc: corrected QT dispersion; QTI: QT prolongation index; SCD: sudden cardiac death.

### The prevalence and outcomes of LQTS in CKD patients (Table 2)

Prolonged QTc is common in patients with CKD [22]. In a study following 6565 participants for a median of 13.3 years, 22.1% (*n* = 1452) had CKD, and 14.5% (*n* = 956) had prolonged QTc. Prolonged QTc was present in 12.9% (*n* = 658) of subjects without CKD and in 20.5% (*n* = 298) of those with CKD (*p* < .0001) [12]. In a population of 154 patients with different stages of CKD, QTc interval prolongation was present in 63.6%. The QTc interval was significantly prolonged across increasing stages of CKD severity. Among patients with stage 2, 3, 4, and 5 CKD, the QTc interval was prolonged in 45%, 59%, 59.3%, and 74.6%, respectively, and the QTc interval was severely prolonged in 15%, 13.6%, 22%, and 23.5%, respectively [3]. In addition, hemodialysis can affect the QT interval, which has been indicated by numerous clinical studies [2,5–7,11,33,34].

The prevalence of all-cause and cardiovascular mortality was significantly increased in CKD patients with aLQTS. Hag et al. observed 280 patients who had been referred for kidney transplant, and 47% died before kidney transplant during the 40-month follow-up period. Patients with a prolonged QTc (39%) had 1-, 3-, and 5-year death rates of 12%, 36%, and 47%, respectively, vs 8%, 24%, and 36% for those with normal QTc, respectively (HR = 1.008, 95% CI: 1.001–1.014, *p* = .016) [10]. Gen et al. observed 122 patients undergoing hemodialysis and prolonged basal QTc was present in 44 patients (36%). The prolonged QTc (HR = 2.16, 95% CI: 1.20–3.91, *p* = .011) interval was an independent predictor of mortality in ESRD regardless of age, sex, diabetes, left ventricular hypertrophy (LVH), left ventricular ejection fraction or the presence of coronary artery disease on angiography [7].

### Causes and mechanisms of QT interval prolongation in CKD

#### Diabetes mellitus

Diabetes accounts for 30–50% of all cases of CKD and affects 285 million (6.4%) adults worldwide [44]. The aLQTS shows a high prevalence in diabetic patients, and it can predict cardiovascular and all-cause death [31,32,34]. The prevalence of aLQTs in diabetic patients is variable from 19% to 44% in different studies. Again both of these studies are overestimating the prevalence of QT prolongation by taking definition as >440 ms, whereas actual definition is >460 ms in females and >450 ms in males [31,32]. The QTc is approximately 11 ms longer in diabetes patients with nephropathy than in those without. Prolonged QTc is a significant marker for the progression of albuminuria in people with diabetic nephropathy. Compared with normoalbuminuria, QTc in patients with microalbuminuria or macroalbuminuria was significantly longer, and QTc was prolonged as urinary albumin excretion increased (normoalbuminuria vs microalbuminuria 425 ± 19 ms vs 435 ± 23 ms, *p* = .01; normoalbuminuria vs macroalbuminuria 425 ± 19 ms vs 442 ± 24 ms, *p* = .0005) [45]. In diabetes, metabolic abnormalities such as hyperglycemia, hyperinsulinemia, hyperlipidemia, and decreased GLP-1 levels can cause activation of the renin-angiotensin system, cardiac autonomic neuropathy, and alterations in calcium homeostasis, leading to myocardial hypertrophy and fibrosis [46]. Abnormalities in metabolism can increase oxidative stress. Oxidative stress is the major metabolic mechanism involved in hERG K^+^ dysfunction, and it can cause a prolonged diabetic QTc interval and action potential duration (APD) [37,46]. In the setting of insulin resistance, a prolonged QTc interval may be related to the defective inactivation of I_CaL_ caused by the decreased protein expression of Cav1.2 and calmodulin [47]. Acute application of insulin concentration-dependently suppressed I_Ks_ currents and led to prolongation of the ventricular APD, which was reflected as QT prolongation on ECG [48].

#### Hypertension

There is a bidirectional relationship between hypertension and CKD. Hypertension can occur as the result of CKD, and it is also an important risk factor for CKD progression. Myocardial hypertrophy induced by hypertension can result in reduced repolarization reserve. The prevalence of myocardial hypertrophy is between 16% and 31% in CKD [29]. Adeseye et al. assessed 210 subjects, of which 140 were new-onset hypertension patients and 70 constituted the control group. The proportion of QT prolongation was 52.14% in the hypertension group and 21.43% in the control group (*p* < .05). The definition of prolong QT interval is >460 ms in female and >450 ms in males. However, this study uniformly defined QT prolongation for men and women was 440 ms, which undoubtedly overestimated the number of patients with prolonged QT interval in hypertensive [30]. The definition of QT prolongation is different in different studies, so the estimated prevalence of QT prolongation in hypertension is also different. But it is undeniable that hypertension is a significant risk factor for prolonged QT [49–52]. The prolongation of QT interval in hypertension is affected by various factors such as cardiomyocyte hypertrophy, changes in the autonomic nervous system and acute alterations in blood pressure (BP) [50,52,53]. QT interval was prolonged in hypertensive subjects with LVH, which may be explained by the heterogeneity of ventricular repolarization in greater ventricular mass [51]. The prolonged QT interval also existed in patients without LVH. Someone thought the QT prolongation was related to increased left ventricular mass (LVM) which existed prior to the development of LVH. However, it is still unclear when hypertension lead to LVM and QT prolongation begin to manifest [50,51]. The downregulation of several K channels responsible for repolarization could contribute to the prolongation of APD in cardiac hypertrophy [54]. The changes of the autonomic nervous system and acute alterations in BP were associated with the QT prolongation in hypertension. However, the mechanism needs further study [50,52].

#### Heart failure

The prevalence of LQTS in patients with heart failure is 60–70.2% [27,28]. Abnormalities of left ventricular function are common in people with CKD, and the incidence increases with the decline in kidney function [55]. Heart failure (HF) in people with CKD is mainly caused by pressure load, volume load and CKD-related nonhemodynamic factors. Nonhemodynamic factors associated with CKD include hyperkalemia, neuroendocrine disorders, and accumulation of toxic metabolites [56]. The potential for dysfunctional I_Kr_ and I_Ks_ [57] and the decay of I_Ca-L_ in HF prolong the plateau of APD. In addition, the Na^+^/Ca^2+^ exchanger (NCX) also plays a prominent role in modulating the APD in myocytes in failing hearts [58]. Late I_Na_ is an integral part of the sodium current, which persists long after the fast-inactivating component and could contribute to the prolongation of the APD [59]. Late I_Na_ is significantly increased in myocardial cells from failing human hearts and selected inhibition of late I_Na_ is effective in shortening the QT interval and reducing ventricular arrhythmogenic activity [60]. The dysfunction of these ion channels may also explain why people with HF are more susceptible to drug-induced LQTS than normal individuals. For example, the incidence of TdP caused by ibutilide was between 5.9% and 11.4% in patients with left ventricular dysfunction and between 1.7% and 4.1% in normal individuals [61].

#### Uremic toxins

Uremic retention compounds with harmful biological or biochemical activity are defined as ‘uremic toxins’, which include urea, creatinine, phosphorus, parathyroid hormone (PTH), fibroblastic growth factor-23 (FGF23), indoxyl sulfate (IS) and homocysteine [62]. Multiple linear regression analysis revealed that serum uric acid was significantly associated with both HR and QTc (*p* = .0061, *α* = .01) [63]. High concentrations of uric acid can increase inflammatory responses and oxidative stress, which can affect myocardial electrophysiological properties and increase the incidence of arrhythmic events [64]. Hyperphosphatemia is associated with cardiac fibrosis and myocardial hypertrophy, which can prolong the QT interval. The average differences in QT interval duration in adjusted models comparing the highest vs. the lowest quartiles of serum phosphorus were 3.9 ms (95% CI 2.0 ms to 5.9 ms; *p* for trend <.001) in NHANES III study. In ARIC, the average differences in QT interval duration in adjusted models comparing the highest versus the lowest quartiles of total phosphorus were 2.3 ms (95% CI 1.3 ms to 3.3 ms; *p* for trend <.001) [65]. Hyperphosphatemia can stimulate PTH release. Palmeri et al. showed that PTH could prolong phase 2 of the APD, independent of serum calcium levels, both in animals and in patients with coronary artery disease [66]. Evidence from basic research has shown that PTH contributes to four major cardiovascular effects: contractile disturbance, cardiomyocyte hypertrophy, cardiac interstitial fibrosis and the vasodilatory effect [62]. Patch-clamp studies in animal models have demonstrated a modulating effect of PTH on cardiac repolarization through changes in both serum and intracellular calcium concentrations [67]. Low serum klotho levels and high FGF23 levels resulting from high phosphate loading accelerate the progression of LVH, cardiac fibrosis, cardiac mechanical dysfunction and cardiac arrhythmias [62,68,69]. IS can downregulate K_I_ channel activity and increase the APD, further prolonging the QT interval [70,71]. The concentration of serum homocysteine gradually accumulates with the decline in renal function in patients with CKD [72]. Plasma levels of homocysteine and 12-lead ECGs were assessed in a population-based study of 7002 participants (3260 males and 3742 females) 35 years of age and older. They found the mean homocysteine levels among participants who had QTc > 440 ms were higher than those who had QTc ≤ 440 ms (*p* = .031). However, this study showed that the prolonged QT was related to both high and low homocysteine levels [73]. Hyperhomocysteinemia can affect the synchronization of myocardial contraction, reflected by the prolongation of the QRS and QT interval on electrocardiogram. Cardiac remodeling induced by matrix metalloproteinase-2 and matrix metalloproteinase-9 and decreased expression of connexin 40, 43, and 45 appear to play a role in the pathomechanism of QT prolongation in hyperhomocysteinemia [74]. The relationship between homocysteine and QT interval needs further studies.

#### Electrolyte disorder and hemodialysis

Hemodialysis is a therapeutic procedure that uses the extracorporeal circulation of blood to ameliorate the azotemia, fluid, electrolyte, and acid–base abnormalities that are characteristic of uremic syndrome. In dialysis patients, a number of small studies using ambulatory electrocardiography showed that the QT interval was increased in the hemodialysis population compared with that in healthy controls during dialysis [2,5–7,11]. Electrolyte disorder, hypoperfusion and rapid changes in intra- and extracellular electrolytes during dialysis, particularly changes in potassium, magnesium and calcium, might account for the QT prolongation [2,4,5].

Hypokalemia is a risk factor for a prolonged QT interval, and its prevalence in CKD is 12%–18% [75,76]. Low potassium can be caused by low dietary potassium intake, malnutrition, chronic diarrhea, or the use of prescription drugs [77]. Furthermore, with the use of a low potassium dialysate, K^+^ is removed mainly from the extracellular space and only slightly from the intracellular space; thus, the extracellular potassium level decreases too abruptly, and the intracellular/extracellular potassium ratio increases rapidly [78].

Hypomagnesaemia is common in CKD patients and is linked to the increased risk of development of coronary artery disease as well as the major cardiovascular risk factors such as dyslipidemia, endothelial dysfunction, metabolic syndrome, atherosclerosis, diabetes, hypertension and elevated fasting insulin levels, suggestive of insulin resistance [79]. Magnesium is a cofactor for sodium-potassium ATPase activity. By facilitating the influx of potassium into cells, magnesium stabilizes the membrane potential. Hypomagnesemia impairs the function of the sodium–potassium ATPase pump, leading to reduced intramyocyte potassium concentrations [80]. However, a recent review article showed that hypomagnesemia is not a risk factor for prolong QT interval [81]. MgSO_4_ can prevents the recurrence of TdP without changing the QT interval. The mechanism of action of MgSO_4_ is not well understood.

Calcium is crucial for the entirety of the APD. The inward calcium current is a depolarizing current that prolongs the ventricular APD, and it can influence calcium channels and electrogenic exchangers in myocardial cells [65]. CKD can destroy the balance of calcium *in vivo* and can cause a variety of changes in Ca^2+^ regulatory mechanisms. Prevalence of hypocalcemia in CKD patients is high and is strongly correlated with prolong QTc [81]. Calcium treatment in patients with hypocalcemia can significantly shorten the repolarization interval and reduce the number of ventricular premature complexes [82].

Hypertension is highly prevalent and difficult to control in patients with long-term dialysis. Although 89% of these patients undergo treatment, only 38% have adequate control of their hypertension [83]. The ultrafiltration was associate with the change of QT interval before and after HD [71]. However, rapid fluid clearance during hemodialysis may result in a sharp decrease in circulating volume, leading to hypotension, tissue ischemia, cardiac remodeling and arrhythmia [84]. A systematic literature showed the prevalence of intradialytic hypotension was 10.1% and 11.6% for the European Best Practice Guideline (EBPG) definition and the Nadir <90 definition, respectively [85]. The intradialytic hypotension was an independent risk factor for cardiovascular morbidity and mortality in hemodialysis patients [86]. Transient myocardial ischemia caused by intradialytic hypotension can result in myocardial stunning [87]. Furthermore, circulating endotoxin was found to be grossly elevated in stage 3–5 CKD patients, with a further increase in endotoxemia in the HD population, also correlating with the increased severity of myocardial stunning (*R* = 0.44, *p* = .035) [88]. Calcium overload and reactive oxygen species (ROS) generation were two main mechanisms of myocardial stunning [87]. ROS has significant relationship with myocardial fibrosis [89]. Nowadays, myocardial native T1 times on cardiac MRI have been shown to be a surrogate marker of myocardial fibrosis [90]. Elaine et al. found QTc was positively correlated with septal T1 in ESRD patients on hemodialysis (Spearman’s *R* = 0.376, *p* = .045) [91].

#### Autonomic dysfunction

Cardiovascular autonomic neuropathy (CAN) affects both the heart rate and ventricular repolarization. A decrease in heart rate variability and an increase in QTc are the initial signs of CAN. Cardiac sympathetic overdrive decreases vagal control in CKD. The association between CAN and GFR is curvilinear [92]. Autonomic neuropathy is associated with prolonged QT in diabetes, which may be due to the uneven sympathetic innervation of the heart, leading to uneven duration of ventricular depolarization and repolarization. However, there was no significant relationship between autonomic neuropathy and QT prolongation in ESRD [33,35]. The presence of other factors that prolong QT in patients with ESRD may affect the contribution of autonomic neuropathy to QT prolongation. Meanwhile, the effect of autonomic neuropathy on QT prolongation in patients with early stage of CKD has not been studied.

#### Drugs

CKD patients are often prescribed many medications with the potential of prolonging the QT interval [93]. Drug pharmacokinetics are complex in patients with CKD. Renal excretion of many commonly used medications is decreased in CKD which makes these medications to cause much more QT interval prolongation [21]. Kidney transplantation can correct electrolyte abnormalities, reverse myocardial remodeling and improve autonomic nerve function, which may be related to QT interval shortening in CKD patients [94,95]. However, the QT interval was measured only after two weeks, and the effect of immunosuppressive drugs on the QT interval was not assessed [96]. In one study, QT interval prolongation was caused by immunosuppressive drugs (tacrolimus, cyclosporine A, everolimus and azathioprine) in the long-term follow-up of kidney transplant patients [23]. We summarize the drugs known to cause TdP that require dose adjustment in patients with chronic kidney disease (Table 3) [21,22,24–26,97–99].

**Table 3.** Drugs known to cause torsade de pointes that require dose adjustment in patients with chronic kidney disease.

Drugs	References
Antiarrhythmics: Amiodarone; Disopyramide; Dofetilide; Ibutilide; Procainamide; Quinidine; Sotalol; Flecainide	[21,22,97]
Antibiotics: Chloroquine; Ciprofloxacin; Clarithromycin Erythromycin; Halofantrine; Pentamidine; Sparfloxacin; Antipsychotics; Chlorpromazine; Fluconazole; Levofloxacin	[21,22,24,25,97]
Haloperidol: Mesoridazinea; Pimozide; Thioridazine	[21,22,97]
Antinauseants: Domperidone; Droperidol	[21,22]
Antineoplastic: Arsenic trioxide; Vandetanib; Eribulin	[26,98,97]
Gastric promotility: Cisapridea	[21,22,97]
Opiates: Methadone; Levomethadyl	[22,97]
Antihistamines: Terfenadinea; Astemizolea	[21,97]
Immunosuppressive drugs: Tacrolimus; Cyclosporine A; Everolimus; Azathioprine	[93]

CKD: chronic kidney disease; TdP: torsades de pointes.

### Management of aLQTS in CKD

The elimination of QT prolonging risk factors is the cornerstone of aLQTS management. However, the risk factors for QT prolongation are sometimes difficult to avoid, in which case regular ECG monitoring and necessary interventions are important, especially in patients who have had TdP. Interventions with aLQTS with TdP include maintenance of high normal serum potassium levels, intravenous magnesium, isoproterenol infusion, and temporary transvenous ventricular pacing. It is worth noting that different interventions are needed depending on the presence of TdP.

#### QT prolongation without TdP

Typically, the development of severe QT prolongation or TdP requires several precipitating factors to occur in combination to overcome a ‘repolarization reserve’ generated by the presence of multiple cardiac channels. Patients with QT prolongation in the absence of observed TdP episodes should undergo continuous ECG monitoring. The frequency of QT monitoring depends on the clinical situation and the extent of QT prolongation. The necessary interventions need to be performed immediately when accompanied by ECG signs of impending TdP, such as QTc prolongation >500 ms, QT-U prolongation and distortion after a pause, onset of ventricular ectopy and couplets, macroscopic T-wave alternans (TWA), or episodes of polymorphic ventricular tachycardia that are initiated with a short-long-short R-R cycle sequence [100]. Appropriate interventions include removal of risk factors and correcting the electrolyte abnormalities [101]. Magnesium administration does not shorten the QT interval, and the administration of intravenous magnesium to patients with QT prolongation in the absence of TdP is probably unnecessary [102].

#### QT prolongation with TdP

Most TdP can terminate spontaneously. However, for patients with TdP that does not terminate spontaneously or that degenerates into ventricular fibrillation, immediate direct-current cardioversion should be performed [103]. The treatments, such as removal of the offending causes, the maintenance of a high-normal serum potassium level and the use of intravenous magnesium, anti-arrhythmic drugs or cardiac pacing are beneficial for terminating TdP (Table 4) [103–110].

**Table 4.** Treatment of torsades de pointe.

Treatments	Characteristics	Mechanism
Magnesium sulfate	Magnesium sulfate is currently recommended as immediate first line treatment for TdP. In the treatment of TdP, 2 g of magnesium sulfate in adults (25–50 mg/kg up to 2 g in children) is given over 60 seconds. This dose may be repeated in 5–15 minutes for refractory dysrhythmias. Continuous infusion of up to 3–10 mg/min in adults may also be started for persistently refractory dysrhythmias The most prominent adverse effect is flushing, but nausea and vomiting, hypotension and drowsiness can occur with higher doses.	Magnesium may reduce EAD by inhibiting the late calcium influx via L-type calcium channels that are associated with delayed ventricular repolarization. Magnesium stabilize the membrane potential by facilitating potassium influx, correcting dispersed repolarization without shortening the action potential duration.
Potassium	Potassium was always be considered, although there is little evidence to support this practice. Repletion of potassium to levels of 4.5–5 mmol/L.	The potassium is an important adjunct to intravenous magnesium for the short-term prevention of torsade de pointes.
Isoproterenol	Case reports suggest benefit. For refractory TdP, initial dosing of isoproterenol is 0.5–1.0 μg/min in an adult and 0.1 μg/(kg min) in a child. Upper limit of dosing is 20 μg/min in an adult and 1.5 μg/(kg min) in a child. Isoproterenol may be fatal if given to patients with a ventricular tachycardia that is not TdP.	Isoproterenol shortens the QT interval and effective refractory period. Suppress EAD and TdP by enhancing outward K^+^ currents, accelerating heart rate and repolarization, and shortening the action potential duration.
Lidocaine	Case reports suggest benefit.	Lidocaine is Class Ib antiarrhythmic drug. Lidocaine may enhance outward K^+^ currents to short the QT interval. Lidocaine may also partially result from the inhibition of conduction of EAD from the Purkinje network to the myocardium.
Phenytoin	Case reports suggest benefit.	Phenytoin is Class Ib antiarrhythmic drug. The phenytoin include the decrease in ventricular automaticity especially in Purkinje fibers and a central antiarrhythmic effect through decrease in sympathetic discharge and increase in the atrioventricular conduction velocity in addition to blocking calcium-dependent depolarization in the plateau phase of action potential favoring repolarization of Purkinje fibers and preventing EAD and inhibiting EAD conduction from the Purkinje network to the surrounding myocardium.
Atropine	Case reports suggest benefit. Atropine may induce paradoxical bradycardia, increasing the risk of TdP.	Atropine is expected to increase the heart rate, thereby shortening the QTc interval and suppressing the arrhythmia.
Mexiletine	Case reports suggest benefit. Mexiletine may be an effective treatment approach to terminate refractory TdP from several acquired causes of LQTS.	Mexiletine is Class Ib antiarrhythmic drug. Mexiletine is also a potent blocker of I_Na-L_, which is effective in abbreviating repolarization, decreasing dispersion of repolarization, suppressing EAD, and preventing TdP.
Transvenous pacing	Case reports suggest benefit. Refractory TdP may be amenable to cardiac pacing.	A ventricular rate of 90–110 bpm is sufficient to eliminate ventricular ectopy, and some patients may require heart rates as high as 140.

EAD: early after depolarization; LQTS: long QT syndrome; TdP: torsades de pointes.

**Figure 1.**
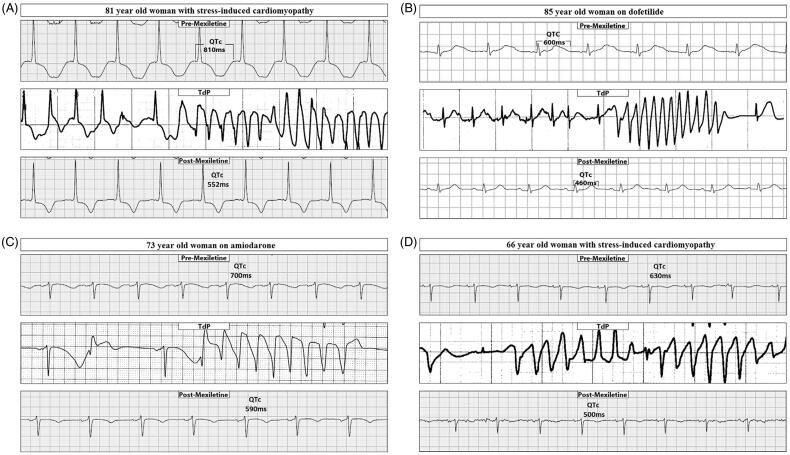
Electrocardiogram (ECG) strips of four patients with long QT premexiletine, followed by torsade de pointes (TdP) and postmexiletine ECG strips. In patient A, the postmexiletine ECG was recorded 24 h after the first dose and after a total administration of 400 mg. In patient B, the post-mexiletine ECG was recorded 15 h after the first dose and after a total administration of 300 mg. In patient C, the postmexiletine ECG was recorded 25 h after the first dose and after a total administration of 600 mg of mexiletine. In patient D, the postmexiletine ECG was recorded 23 h after the first dose and after a total administration of 600 mg. Note that all four episodes of TdP occurred following a long-short interval. (With permission from Xiaolin Xue [109]).

Mexiletine has a significant therapeutic effect on refractory TdP and that it can shorten the QT interval of the basic ECG. The enhanced late sodium channel currents (I_Na-L_) can potentially cause pro-arrhythmic phenotypes [111]. Enhanced I_Na-L_ can be observed in acquired conditions, such as hypertrophic cardiomyopathy, heart failure and drug-induced arrhythmias [112,113]. Mexiletine, a Vaughan-Williams class Ib antiarrhythmic agent, can shorten the APD by selectively suppressing I_Na-L_ without affecting QRS duration [59]. We once reported that 12 patients with refractory TdP secondary to aLQTS were treated with oral mexiletine, 150–450 mg/day orally. In these patients, mexiletine could terminate the acute TdP and shorten the QTc interval (Figure 1) [109].

## Summary

CKD is a growing public health problem. QT interval prolongation is responsible for CVD, especially SCD, which is a major cause of death in the CKD population. Numerous factors such as diabetes mellitus, hypertension, heart failure, electrolyte disorder, uremic toxins, hemodialysis and drugs can influence the repolarization of cardiac cells in CKD. However, there is still a lack of how to improve the dilemma of QT prolongation in patients with kidney disease. Especially like a wide variety of uremic toxins, their effects on myocardial depolarization and repolarization are not well understood. The traditional treatment of aLQTS patients with TdP includes removal of the offending causes, the maintenance of a high-normal serum potassium level and the use of drugs. The management of aLQTS in CKD patients still needs further exploration.
